# Supplementary material for: Nna1, Essential for Purkinje Cell Survival, Is also Associated with Emotion and Memory
Source: Int J Mol Sci. 2022 Oct 26;23(21):12961. doi: 10.3390/ijms232112961 (PMC9654422; doi:10.3390/ijms232112961)
Supplement: Supplementary file 1 [file ijms-23-12961-s001.zip › ijms-1972761-supplementary.pdf]

Nna1, essential for Purkinje cell survival, is also associated with emotion and memory

Li Zhou, Kotaro Konno, Maya Yamazaki, Manabu Abe, Rie Natsume, Masahiko Watanabe,
Hirohide Takebayashi*, Kenji Sakimura *

Figure S1

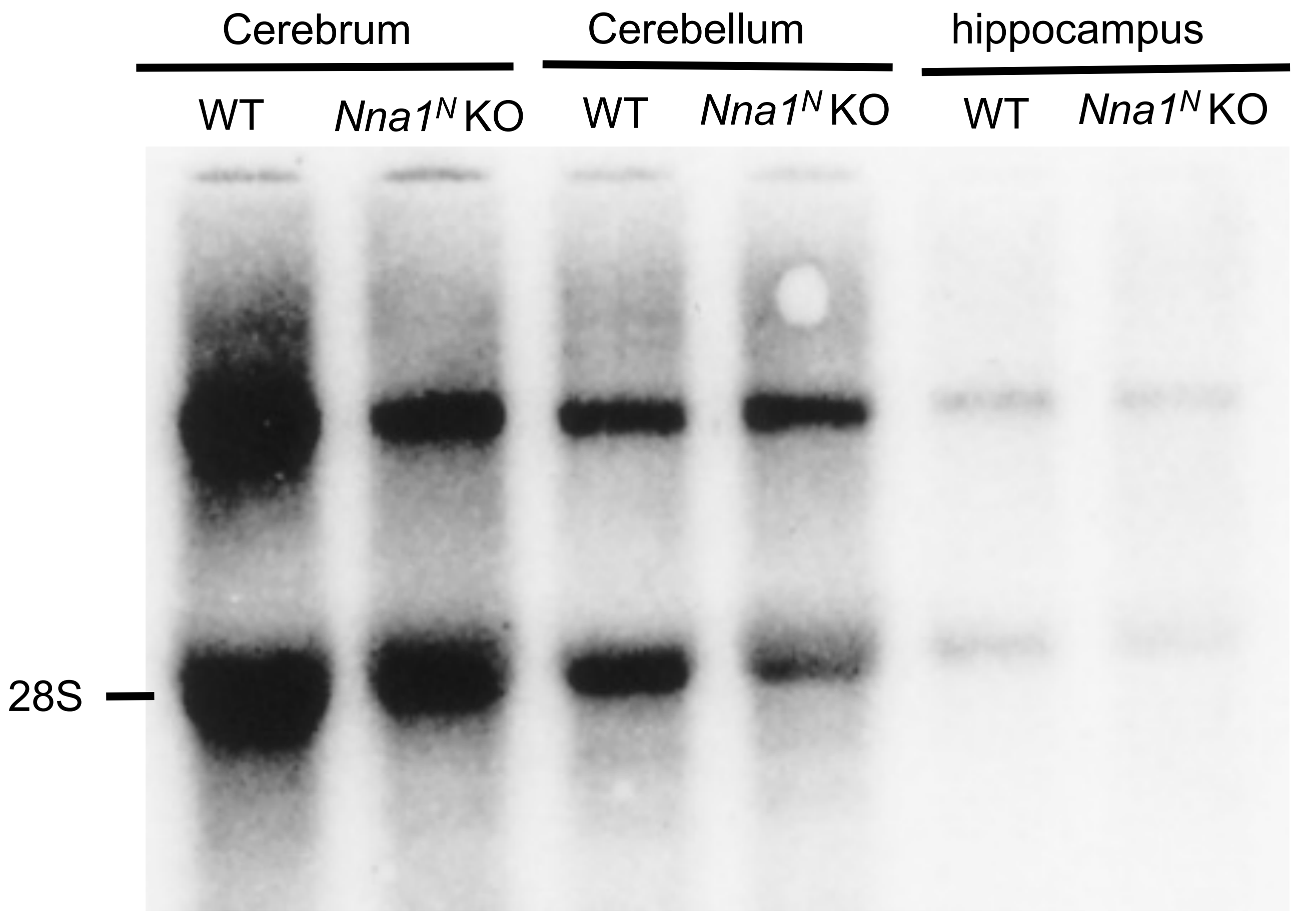

Figure S1. Northern blot analysis of WT and *Nna1*^{N} KO mice in the cerebrum, cerebellum, and hippocampus. Note a mild increase of *Nna1* mRNA from *Nna1*^{N} KO mice in a different part of the brain.

Figure S2

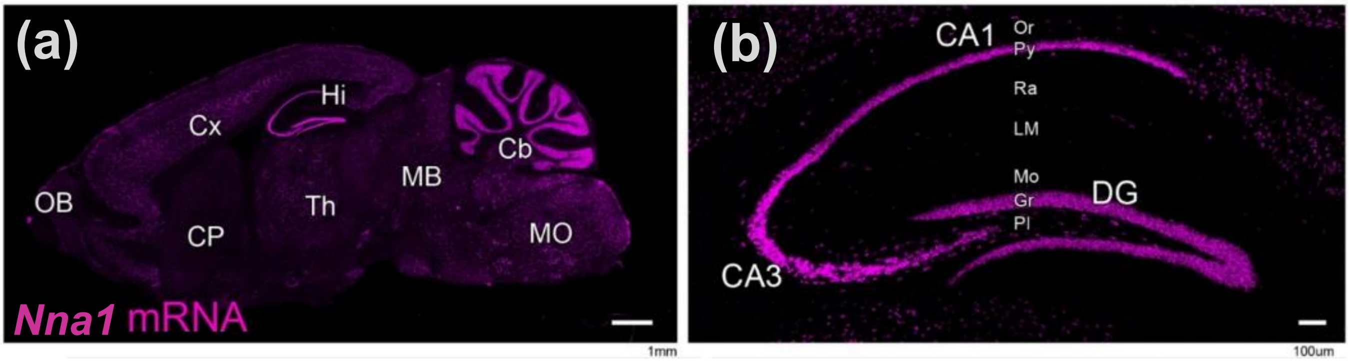

Figure S2. *In situ* hybridization of *Nna1* mRNA on the parasagittal sections of the whole brain. (a) Note the high expression of *Nna1* mRNA in the Purkinje cell layer and granule cell layer of the cerebellum and hippocampus and mild expression in the cortex, thalamus, medulla oblongata, and olfactory bulb. (b) *Nna1* mRNA expression was detected in the CA1–CA3 and DG regions of the hippocampus. Scale bars in (a): 100µm, scale bars in (b): 1mm.

Figure S3

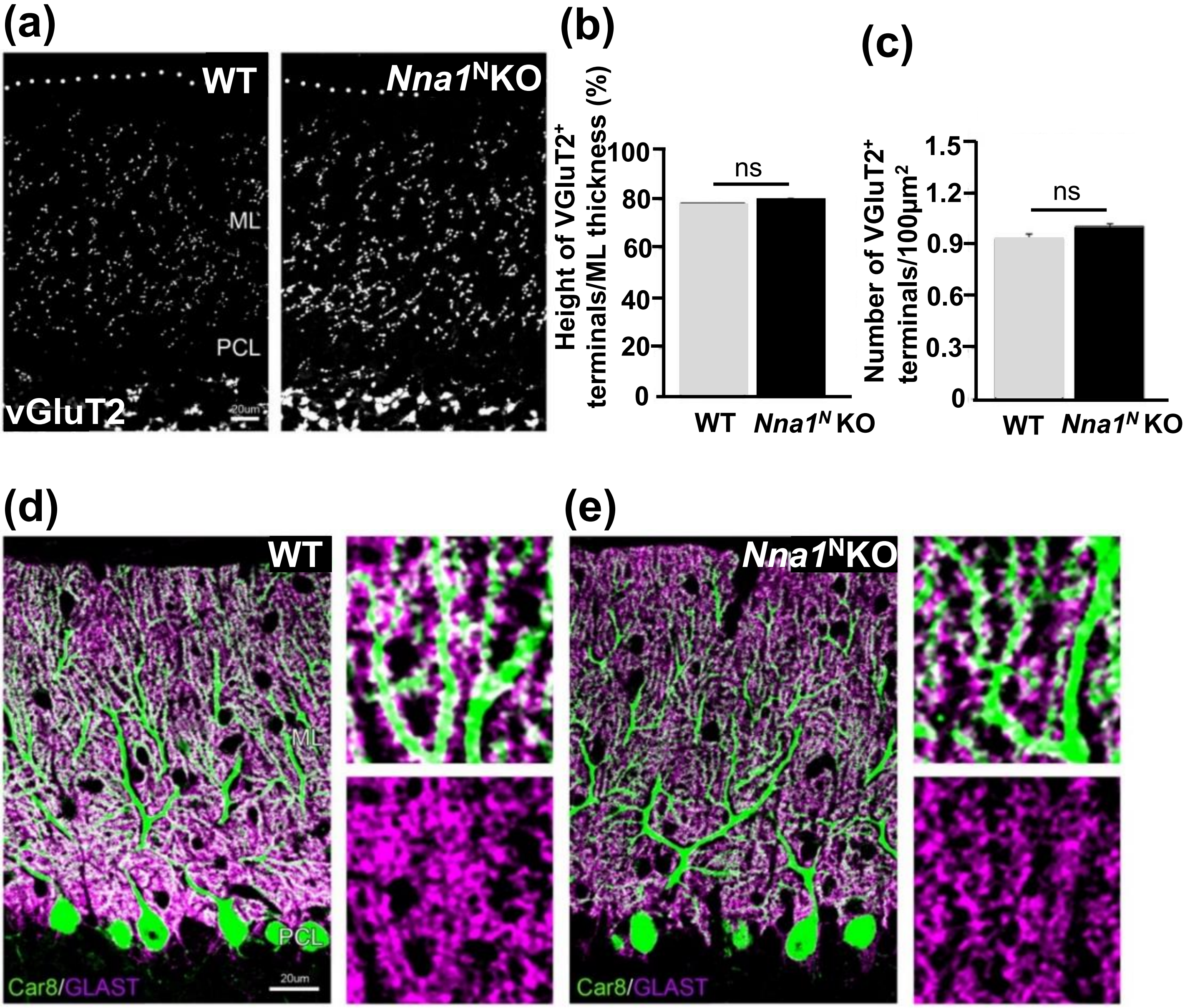

Figure S3. Histological analyses on the cerebellum of *Nna1^N^* KO mice. (a) vGluT2 IHC on the parasagittal sections of WT and *Nna1^N^* KO mice. There was no obvious difference in vGluT2 terminal intensity in the molecular cell layer between WT and *Nna1^N^* KO mice. (b) Height of VGluT2+terminals in the height of the molecular layer. (c) Number of VGluT2+terminals in the molecular layer. There was no significant difference between WT and *Nna1^N^* KO cerebellum. (d,e) Double IHC of Car8 (green) and GLAST (magenta) in the cerebellum of WT (d) and *Nna1^N^* KO mice (e). Scale bars: 20μm.

Figure S4

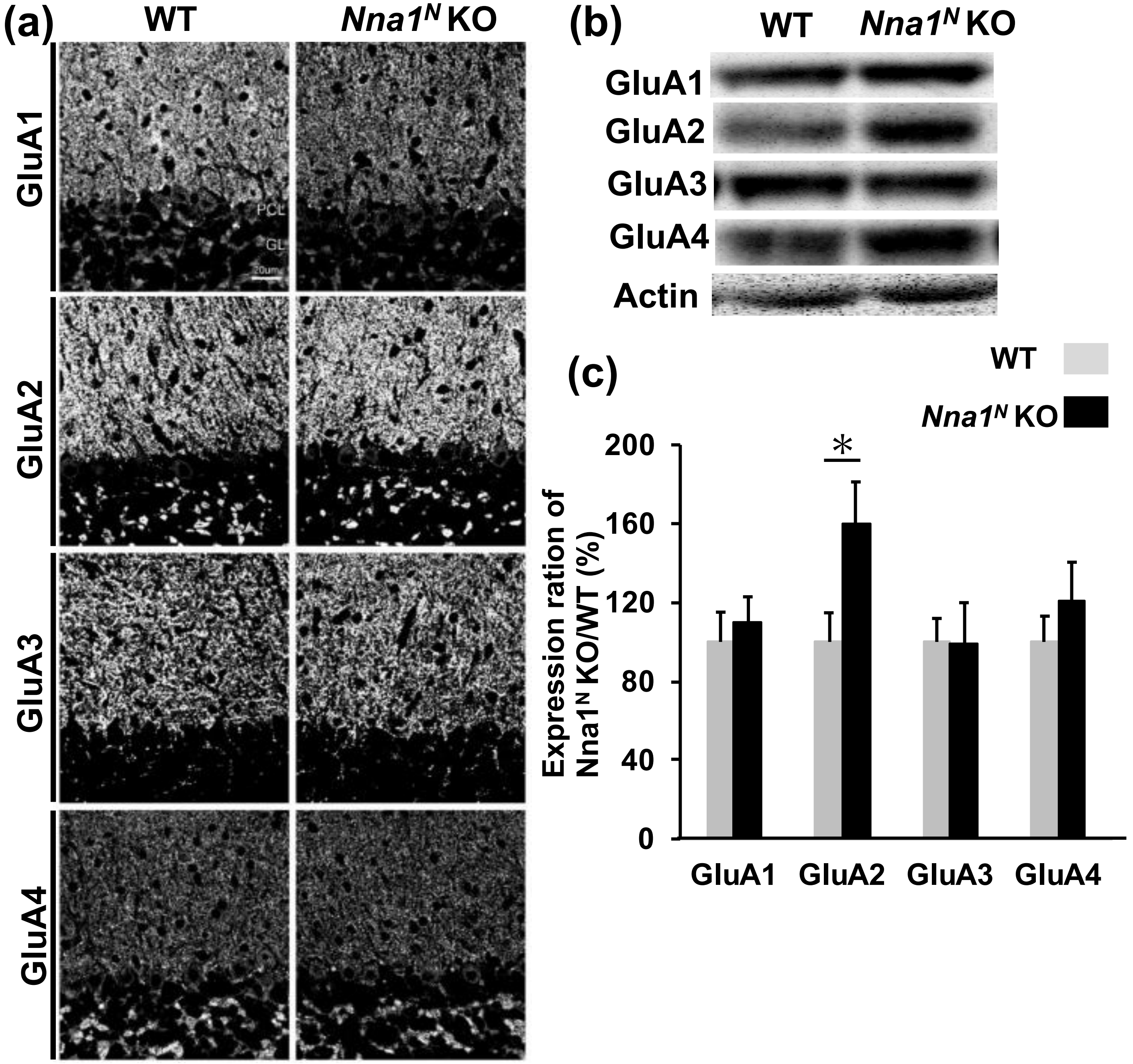

Figure S4. Expression of AMPA receptors in the cerebellum by immunohistochemistry and western blotting analysis. (a) No significant difference in the expression of GluA1, GluA3, or GluA4 was observed between *Nna1*^{N} KO and WT cerebellum; only a mild GluA2 increase was observed in *Nna1*^{N} KO mice. (b) Western blotting using antibodies against GluA1, GluA2, GluA3, and GluA4. Crude fractions prepared from the cerebellum were loaded 20μg each lane. (c) Quantification of western blotting data. Note that increased GluA2 expression was observed in the *Nna1*^{N} KO mice. *$p < 0.05$, Student's *t*-test. All values presented are mean ± SEM from 3 experiments.
